# Teachers’ perceptions of classroom climate and wellbeing: the role of physical classroom conditions in Chile

**DOI:** 10.3389/fpsyg.2025.1567464

**Published:** 2025-09-25

**Authors:** Flavio Muñoz-Troncoso, Enrique H. Riquelme

**Affiliations:** ^1^Faculty of Education, Universidad Católica de Temuco, Temuco, Chile; ^2^Faculty of Social Sciences and Arts, Universidad Mayor, Temuco, Chile

**Keywords:** classroom climate, school infrastructure, teacher perceptions, personal wellbeing, Chilean education system, confirmatory factor analysis and CFA, structural equation modeling and SEM

## Abstract

**Introduction:**

Classroom climate has gained relevance as concerns have grown about its deterioration and its impact on both academic work and the daily lives of school communities. Different approaches have tried to explain this multivariate problem, yet few have considered the workspace itself as a factor that also explains the phenomenon. This study explores how physical classroom conditions influence perceived classroom climate among Chilean teachers, addressing a gap in research in the context of educational inequalities between public and private schools.

**Methods:**

Within the framework of a quantitative, non-experimental and cross-sectional design, 6,038 teachers of different ages and genders participated. Scales from Chile’s JUNAEB program were adapted to measure classroom climate and personal well-being, both using a Likert-type response format.

**Results:**

The first-order model showed that the physical conditions of the classroom significantly and positively affect key latent variables, such as personal well-being (coefficient of 0.502) and teacher-student relationships (coefficient of 0.699). The model demonstrated good fit indices (X^2^ = 7,972.987, RMSEA = 0.061, CFI = 0.953), which supports the relevance of these relationships.

**Discussion:**

Key physical aspects such as space, lighting, and temperature were found to directly affect teachers’ perceptions of classroom climate, with implications for students’ emotional and academic outcomes. The study concludes by examining classroom infrastructure and resources as elements to consider when seeking to improve both personal well-being and classroom climate, ultimately fostering inclusive and effective learning environments.

## Introduction

School violence is a multifaceted phenomenon rooted in the interplay of individual, family, and societal factors, each deeply tied to emotional and psychological wellbeing. On an individual level, psychological challenges such as anxiety, depression, and aggression, combined with a fascination for violence or weapons, and low resilience, significantly increase the likelihood of violent behaviors (Moon and Lee, 2020; Timm and Aydin, 2020). Resilience, which reflects the ability to adapt to adversity, is particularly crucial. Students with low resilience struggle to regulate emotions and cope with stress, making them more vulnerable to both perpetrating and experiencing violence. Addressing emotional vulnerabilities and fostering coping mechanisms could serve as a cornerstone in mitigating violence at its root. Family influences also play a pivotal role in shaping emotional responses related to violence. Exposure to domestic violence, where children witness aggression as a normative conflict resolution strategy, creates a blueprint for violent behavior. Ineffective parenting styles, such as overly authoritarian or neglectful approaches, fail to provide the emotional security necessary for healthy development (Lawrence, 2022; Solimannejad et al., 2022). Economic stressors within families exacerbate these dynamics, leading to heightened emotional distress in children, which may manifest as aggression or withdrawal in school settings. The emotional climate within the family directly influences how young people perceive themselves and others, often dictating their responses to conflict or provocation.

On a societal level, peer pressure, cultural diversity, and pervasive exposure to media violence compound the problem. Peer dynamics, particularly in adolescence, are often driven by the need for acceptance, leading some students to engage in aggressive behaviors to gain social standing or avoid victimization (Moon and Lee, 2020). The media’s normalization of violence, from movies to video games, desensitizes young minds and distorts emotional responses to aggression, contributing to diminished empathy and increased acceptance of violent behavior. Cultural diversity, while enriching, can also lead to misunderstandings or tensions in schools, particularly when students are not equipped with the emotional intelligence or cultural sensitivity needed to navigate differences.

Within the school environment itself, structural and relational factors exacerbate the problem. Academic apathy, often stemming from uninspiring curricula or a lack of emotional connection between students and their studies, fosters disengagement, which can escalate into disruptive or violent behaviors. Inefficient school environments, characterized by overcrowded classrooms, poor infrastructure, or an absence of emotional support systems, create fertile ground for conflict (Solimannejad et al., 2022). Teacher attitudes also play a critical role; negative or dismissive interactions between teachers and students can contribute to feelings of alienation and resentment. When students lack positive role models or emotional validation within the school, their likelihood of engaging in aggressive behaviors increases.

These interconnected factors contribute to various forms of school violence, ranging from subtle harassment and bullying to overt physical aggression (Zhang and Jiang, 2022). The emotional toll of such experiences is profound, affecting not only the victims but also the perpetrators and bystanders. Longitudinal analyses reveal that school violence has long-lasting consequences, including deteriorated mental health, diminished academic performance, and an increased risk of criminal involvement later in life (Polanin et al., 2021). Victims often experience heightened anxiety, depression, and feelings of isolation, while perpetrators may struggle with guilt, anger, or an inability to form healthy relationships in adulthood.

Addressing school violence requires comprehensive, emotionally informed approaches, equipping students with the emotional tools to navigate stress, conflict, and adversity; furthermore, positive parenting programs can help families create nurturing environments that foster emotional security and healthy conflict resolution (Mayer et al., 2021). Within schools, prevention strategies should prioritize cultivating emotionally supportive environments, promoting empathy through social–emotional learning (SEL) programs, and encouraging positive teacher-student relationships. Research emphasizes that schools with proactive emotional support systems—such as counseling services, peer mediation programs, and inclusive practices—see significant reductions in violence and improvements in the overall school climate (Taylor et al., 2017). Ultimately, school violence is not just a behavioral issue but a deeply emotional one, rooted in the inability to process and regulate emotions effectively. By addressing the emotional underpinnings of violence at individual, family, and societal levels, we can create safer, more nurturing schools where all students feel valued and supported.

School violence has profound effects on students’ academic performance, mental health, and overall wellbeing. Forms of violence such as direct aggression, discrimination, and cyberbullying significantly reduce academic performance (Polanin et al., 2021; Bravo-Sanzana et al., 2022). Victims also report reduced life satisfaction and increased emotional challenges (Liu et al., 2020). Protective factors, such as self-efficacy, educational aspirations, and strong teacher-student relationships, can mitigate these effects (Bravo-Sanzana et al., 2022). Emotional regulation, in particular, improves wellbeing and academic engagement (Eriksen and Bru, 2023). Despite their influence, schools generally have a limited impact on student wellbeing (Govorova et al., 2020). Promoting social–emotional education and fostering positive classroom climates remain crucial to addressing these challenges (Mayer et al., 2021).

School climate analysis has historically been approached from a perspective focused on interpersonal and normative factors, such as student relationships, discipline, teacher support, and social interactions within the classroom. Indeed, Teacher support, student engagement, and classroom affiliation are critical elements (Jiménez et al., 2021; Montero and Saltos, 2021). Faculty-student relationships and perceptions of academic competence indirectly influence violence and victimization through their effect on classroom climate (Jiménez et al., 2021). Cooperative environments also foster positive attitudes toward diversity (Barksdale et al., 2021; Miklikowska et al., 2021; Cheon et al., 2022; Wachs et al., 2023).

This approach has contributed significantly to identifying how a positive school climate can prevent conflicts, foster prosocial attitudes, and improve academic outcomes. For example, research such as that conducted by Cheon et al. (2022) has shown that creating a supportive teaching environment, characterized by autonomy and positive reinforcement, reduces antisocial behaviors among students and promotes greater participation in school activities. Similarly, Jiménez et al. (2021) highlighted that a school climate based on positive relationships between teachers and students acts as a mediator in the prevention of victimization and school violence, while improving academic performance.

This traditional approach, while valuable, has overlooked the influence of physical and structural classroom factors on school climate. Elements such as lighting, temperature, available space, and classroom design are components of the so-called Physical Workspace, which, while having a direct impact on learning and emotional regulation, have received less attention in school climate research. Recent studies have begun to address this gap. For example, Brink et al. (2021) demonstrated that environmental conditions in classrooms, such as air quality, noise levels, and lighting, directly affect students’ academic performance and teachers’ wellbeing. These physical conditions not only influence comfort levels, but also students’ ability to focus and regulate their emotions, which are essential for a positive classroom climate.

In this study, the physical classroom environment is understood as a set of interrelated elements identified in international and regional research as determinants of learning and teacher wellbeing. These include spatial conditions and furniture arrangement, natural and artificial lighting, ventilation and temperature, as well as safety, cleanliness, and access to essential facilities and resources (Duarte et al., 2011; Barrett et al., 2013; Boix-Vilella et al., 2021; Brink et al., 2021). Deficient conditions in these areas have been shown to undermine students’ concentration and emotional self-regulation, while increasing stress and reducing teachers’ job satisfaction (Eriksen and Bru, 2023; Siddique et al., 2023). In this study, this broader framework was operationalized through indicators that reflect teachers’ direct perceptions of their working conditions. These indicators refer to having sufficient space to conduct lessons, adequate classroom acoustics, cleanliness and order, and the overall sense of comfort and satisfaction associated with working in the classroom. Such dimensions are in line with international evidence that highlights the role of spatial adequacy, environmental quality, and maintenance in shaping both teaching effectiveness and educational outcomes (Barrett et al., 2018; Siddique et al., 2023; Espinosa-Andrade et al., 2024).

In addition, a suitable physical environment can serve as a facilitator of social and emotional interactions, Eriksen and Bru (2023) argue that a structured and safe space within the classroom fosters students’ emotional self-regulation, which, in turn, improves their willingness to participate in collaborative activities and reduces the incidence of interpersonal conflicts, Conversely, classrooms with poor physical conditions can exacerbate stress and perceptions of disorganization, negatively affecting both relational dynamics and learning (Boix-Vilella et al., 2021). The classroom climate is essential to prevent conflicts and promote positive results. It has been shown that supportive and cooperative environments reduce antisocial behavior, improve academic success, and improve social skills (Wang et al., 2020; Cheon et al., 2022).

The right space, facilities, and resources have a positive impact on teacher morale and perceptions of student behavior (Lawrent, 2020; Boix-Vilella et al., 2021). Collegial collaboration in lesson planning improves teaching conditions (Nordgren et al., 2021). Emotional Support During Classroom Interactions Improves Student Engagement (Havik and Westergard, 2020). Factors such as air temperature, lighting, and indoor environmental quality affect teaching effectiveness and academic outcomes in the short term (Brink et al., 2021). While quantitative findings on classroom climate and academic performance vary (Barksdale et al., 2021), qualitative evidence underscores the importance of relationships, classroom organization, and safety in student learning. The positive influence of classroom climate on outcomes remains significant, although moderate (Wang et al., 2020). For all the reasons mentioned, it is recognized that classroom infrastructure and physical space conditions have a significant impact on the perception of classroom climate, as well as on students’ academic and emotional outcomes. However, there is a notable lack of research focused specifically on how teachers perceive the impact of these physical factors on classroom climate within the Chilean context. This knowledge gap is particularly relevant in an education system characterized by inequalities in resources and infrastructure between public and private schools.

Beyond interpersonal and normative factors, recent evidence points to the importance of physical classroom environments in shaping both teaching and learning outcomes. In Chile, instruments for assessing classroom climate have been validated and used to improve school management, although these studies have focused on students rather than teachers (López et al., 2018). International findings show consistent links between infrastructure and educational results: teachers’ perceptions emphasize the relevance of resources and facilities for creating supportive conditions (Siddique et al., 2023), while studies in Ecuador and other Latin American countries report significant associations between infrastructure and student achievement (Duarte et al., 2011; Espinosa-Andrade et al., 2024). Global reviews add that factors such as lighting, ventilation, temperature, and access to specialized learning spaces affect both student performance and teachers’ wellbeing (Barrett et al., 2018). Together, these studies position the physical dimension of schooling as a central factor in understanding classroom climate, especially in contexts of infrastructural inequality. Still, little is known about how teachers themselves perceive these conditions and how their perceptions relate to classroom climate and personal wellbeing.

Therefore, it is critical to investigate how Chilean teachers’ perceptions of the physical space conditions of their classroom influence their assessment of classroom climate.

## Materials and methods

The methodology employed in this study follows a quantitative, non-experimental, cross-sectional design, which allows for the systematic collection and analysis of data at a single point in time without manipulating variables (McMillan and Schumacher, 2005; León and Montero, 2015). This approach is particularly suited for identifying patterns, relationships, or trends among variables within a specific population, offering a robust framework for addressing the research objectives.

The data for this study were collected by the National Board of School Aid and Scholarships (JUNAEB), a Chilean institution that implements public policies focused on the wellbeing and development of students. It should be noted that the data provided by JUNAEB correspond to the 2018 application of the instruments, since datasets from subsequent years have not yet been fully anonymized. As part of its work, the School Coexistence Monitoring Program aims to evaluate and promote a positive and healthy school environment, identifying potential issues such as bullying, interpersonal conflicts, and other forms of violence that impact the emotional wellbeing of students.

The information was delivered to the investigators in response to a formal request submitted within the framework of the Law on Transparency for the Public Function and Access to Information of the State Administration –Law No. 20,285– (Ministerio Secretaría General de la Presidencia, 2008), as specified in exempt resolution DN-02620/2024. This resolution explicitly authorizes access to the requested data, ensuring compliance with Chilean legal frameworks. The dataset provided by JUNAEB was fully anonymized, with all personal identifiers removed prior to delivery. The researchers did not have access to any confidential or identifying information, ensuring strict adherence to ethical standards and privacy protection. JUNAEB confirmed that participation in the survey was voluntary, and participants were informed about the confidentiality of the data and the objectives of the study through an informed consent process.

The use of open government data (OGD) in this study aligns with its recognized potential to contribute to societal and scientific advances (Quarati, 2023). The ethical use of these datasets, anonymized to safeguard the privacy of participants, reinforces the value of leveraging publicly accessible information for research under legal and transparent procedures. To ensure compliance with ethical and legal standards, the study adhered to international guidelines, including the Declaration of Helsinki and the Declaration of Singapore. In addition, it complied with Chilean regulations on the protection of personal data and handling of sensitive information, as stipulated in Law No. 19,628 on the Protection of Private Life and Law No. 20,120 (MINSAL, 2006). These measures ensured the ethical use of data and maintained the transparency and integrity required for scientific analysis. To improve transparency, the resolution authorizing access to data (DN-02620/2024) is described in the Data Availability section of this manuscript.

### Participants

In total, 6,038 teachers from the Chilean school system participated, belonging to age groups ranging from 26 years to 61 + years; 27.4% were men and 72.6% women. This gender distribution is consistent with national statistics on the teaching workforce in Chile (Centro de Estudios Mineduc, 2018). The inclusion criteria were to be a teacher, work in the classroom and have 1 or more years of experience. Although the JUNAEB instruments also include school assistants, only teachers were considered in this study. It should be noted that the data collection reports on the age of the participants at 5-year intervals. This approach, widely used in educational and social research, aims to protect the confidentiality of participants, improve the robustness of statistical analyses by consolidating sample sizes, and facilitate the interpretation of results, thus enabling the effective identification of patterns and trends (Dyrting et al., 2022). Age was reported according to the age ranges shown in Table 1.

**Table 1:** Age ranges.

Age range	Frequency	Percentage	Cumulative percentage
26–30	1,092	18.1	18.1
31–35	1,214	20.2	38.3
36–40	1,081	17.9	56.2
41–45	671	11.1	67.4
46–50	489	8.1	75.5
51–55	619	10.3	85.8
56–60	565	9.4	95.1
61+	293	4.9	100.0
Total	6,024	100.0	

Source: Authors.

### Instrument

The instruments used in this study are two of the three questionnaires developed for the School Coexistence Monitoring Program of the National Board of School Aid and Scholarships (JUNAEB, 2019).

#### Climate in the classroom

This scale, composed by 19 items explore wellbeing within classroom dynamics, including perception of (a) Physical and Psychological Environment (PPE) Refers to the physical and emotional environment in which students learn. This includes aspects such as school infrastructure, safety, cleanliness, and the emotional atmosphere, such as the perception of support and respect within the classroom, example: “I like working in this room”; (b)Teacher-Student Relationships (TSR) Relates to the quality of interactions between teachers and students, including factors such as communication, emotional support, trust, and mutual respect. These relationships are fundamental to students’ wellbeing and academic success; item example: “In this class, each of the students feels accepted and valued as they are”; (c) Learning Opportunities (LO), refers to the opportunities students have to learn and grow within the school environment. This includes the quality of teaching, access to educational resources, and the promotion of meaningful learning experiences; item example: “There are effective mechanisms to support students who have learning disabilities”; (d) Student–Student Relationships (SSR), Refers to the relationships among students, including friendship, mutual respect, cooperation, and conflict resolution, Item example: “In this class, each of the students feels accepted and valued as they are”; (e) Cooperation (COP) Represents the level of collaboration and teamwork among students, as well as their ability to work together toward common goals in an environment of respect and mutual support; item example: “The opinions of parents and guardians on the education of their children are taken into account.”

#### Personal wellbeing

This single-factor questionnaire captures teachers’ perceptions of their motivation to teach and their overall job satisfaction; including their emotional, mental, and physical health, as well as their perception of happiness and satisfaction within the school environment. Includes 10 items that address factors associated with professional performance and the work environment. Item example: I feel good and comfortable at school.

All items from both instruments are measured on a 4-point Likert scale, with higher scores indicating more favorable perceptions.

JUNAEB did not provide information on the validity or reliability of the scales, so the present study adopts the structure as shown in Tables 2, 3. However, it should be acknowledged that the classroom climate scale has been validated in a previous study with Chilean students (López et al., 2018), which reported adequate reliability and construct validity. In the present study, the same instrument is applied to teachers, a population for which no published validation studies were found. It should be noted that, in this research, a structure will be adopted that integrates both questionnaires in the same model.

**Table 2:** Structure of the climate scale in the classroom.

1st order factor	Included articles	Number of articles
Physical environment perception (PPE)	CA01–CA04	4
Teacher-student relationship (TSR)	CA05–CA06	2
Student–student ratio (SSR)	CA07–CA12	6
Learning orientation (LO)	CA13–CA16	4
Organization and participation in the course (COP)	CA17–CA19	3

Source: Authors.

**Table 3:** Structure of the personal wellbeing scale.

Factor	Included items	Number of articles
Personal wellness (PW)	BP01–BP09	9

Source: Authors.

### Analysis plan

To address missing data, cases with more than 20% missing responses were excluded, following a stringent criterion established by the research team with reference to Tabachnick and Fidell (2019). This decision aimed to minimize the proportion of imputed data and preserve as much directly observed information as possible. Missing data patterns were then analyzed using Little’s MCAR Test (Little and Rubin, 2020), along with tests for multivariate normality and homoscedasticity. Since the data were not missing completely at random (*p* < 0.05) and assumptions of normality and homoscedasticity were not met, the missForest imputation method (Stekhoven and Bühlmann, 2012). Based on the measurement models used, a scale is proposed that is examined using Confirmatory Factor Analysis (CFA). This method is essential for validating the structure of theoretical constructs and ensuring that the measures align with the researcher’s understanding of the nature of those constructs (Brown, 2014; Kline, 2023). To assess normality assumptions, the Kolmogorov–Smirnov test is applied, which helps determine whether the distribution of data for all indicators meets the normality criterion. This test is crucial, as CFA assumes that the observed variables follow a multivariate normal distribution, which is critical for the validity of the results obtained (Muthén and Muthén, 2017).

In the context of CFA, model fit indices are evaluated, including the X^2^/df ratio, RMSEA (root mean square error of approximation), CFI (comparative fit index), and TLI (Tucker-Lewis index). These indices are key indicators of the quality of the model’s fit to the observed data. An RMSEA value below 0.06, along with CFI and TLI values above 0.95, is generally considered indicative of a good model fit (Hu and Bentler, 1999; Schreiber et al., 2006). Proper interpretation of these indices is fundamental for validating the proposed model structure and ensuring that the inferences drawn are robust and reliable.

To evaluate convergent validity, the factor loadings of the items on each latent variable are examined, with the expectation that the indicators demonstrate loadings greater than 0.5 and are statistically significant (Hair et al., 1999). This assessment is critical, as it indicates that the items effectively capture the underlying construct they are intended to measure. Following this, the reliability of the factors is calculated using the Composite Reliability (CR) coefficient and the Average Variance Extracted (AVE), adhering to the criteria established by Fornell and Larcker (1981). Specifically, an AVE greater than 0.5 suggests good convergence, while a CR exceeding 0.7 indicates adequate internal reliability.

To establish discriminant validity, the square root of the AVE for each latent variable is compared with the correlations between that factor and others. This comparison ensures that each construct is distinct and not merely a reflection of other variables (Fornell and Larcker, 1981). If the correlations between factors are notably high, a second-order factor model may be evaluated to group the first-order scales under a general construct. This hierarchical factor structure not only facilitates the interpretation of results but also implies a rejection of discriminant validity between factors (Varela et al., 2006).

A Structural Equation Modeling (SEM) approach is then implemented to evaluate the influence of the Perception of the Physical Environment (PPE) scale on the other latent variables. The SEM analysis incorporates the model fit indices used in the CFA, as well as the statistical significance of the gamma parameter (*γ*), which helps determine the magnitude and impact of structural relationships. This comprehensive approach ensures that the relationships among the constructs are accurately represented and understood, providing valuable insights into the dynamics of the model (Hair et al., 2018, 2020). Moreover, it is essential to consider the implications of these findings in the context of existing literature. The integration of convergent and discriminant validity assessments not only strengthens the theoretical framework but also enhances the practical applicability of the constructs in real-world scenarios (Bagozzi and Yi, 1988). By ensuring that the constructs are both reliable and valid, researchers can confidently draw conclusions and make recommendations based on their findings.

## Results

As a result of the missing data handling and imputation process, the final dataset comprised 6,038 teachers, which was the sample used in the subsequent analyses. The proposed first-order model demonstrates a good fit to the data, as indicated by the following indices: X^2^ = 7,972.987, DF = 335, and *p* < 0.001. Although the chi-square value is significant, this is expected in large samples due to its sensitivity to sample size. Therefore, other indices are considered more robust for evaluating model fit. The RMSEA (Root Mean Square Error of Approximation) is 0.061, which falls within the acceptable range (≤0.08) and is close to the threshold for a good fit (≤0.06). Additionally, the CFI (Comparative Fit Index) = 0.953 and TLI (Tucker-Lewis Index) = 0.947 exceed the 0.90 threshold, indicating excellent model fit. These results confirm that the model is appropriate for analyzing the relationships between latent variables.

Regarding the reliability and validity indices presented in Table 4, the results show that the factor loadings of the items range from 0.648 to 0.906, exceeding the minimum threshold of 0.5, which indicates that the items adequately represent their latent constructs. Furthermore, the composite reliability (CR) values for all latent variables are above 0.7, ranging from 0.855 to 0.960, confirming the internal consistency of the scales. On the other hand, the average variance extracted (AVE) values range from 0.597 to 0.766, with most exceeding the 0.5 threshold, providing evidence of good convergent validity.

**Table 4:** First-order reliability and validity indices—climate in the classroom.

Factor	Loads									
	Min	Max	AVE	CR	PPE	TSR	LO	SSR	COP	PW
PPE	0.648	0.868	0.611	0.861	0.781					
TSR	0.843	0.906	0.766	0.867	0.628	0.875				
SSR	0.772	0.867	0.665	0.923	0.699	0.878	0.816			
LO	0.691	0.866	0.597	0.855	0.621	0.775	0.881	0.773		
COP	0.823	0.856	0.704	0.877	0.588	0.707	0.807	0.812	0.839	
PW	0.706	0.898	0.727	0.960	0.502	0.742	0.767	0.777	0.726	0.853

Source: Authors.

However, the discriminant validity analysis reveals high correlations between some latent variables, and not all meet the discriminant validity criterion (i.e., the square root of the AVE for each construct should be greater than its correlations with other constructs). This suggests possible conceptual overlap between certain variables, such as between Teacher-Student Relationship (TSR) and Student–Student Relationships (SSR), as well as between Physical Environment (PPE) and TSR. These correlations may reflect the interconnected nature of these variables in the classroom context but also highlight areas that could benefit from further conceptual and methodological refinement. The first-order model presents solid fit indices and evidence of reliability and convergent validity, although challenges related to discriminant validity persist.

For the second-order model, the proposed model also demonstrates a good fit to the data (X^2^ = 7,960.569; DF = 344; *p* < 0.001; RMSEA = 0.061; CFI = 0.953; TLI = 0.948). The reliability and convergent validity indices are presented in Table 5.

**Table 5:** Second-order reliability and validity indices—climate in the classroom.

Factor	2nd order	Loads			
	Loads	Min	Maximum	AVE	CR
PPE	0.674	0.648	0.868	0.611	0.861
TSR	0.891	0.843	0.906	0.766	0.867
SSR	0.958	0.773	0.866	0.665	0.923
LO	0.931	0.691	0.866	0.597	0.855
COP	0.863	0.823	0.856	0.704	0.877
PW	0.812	0.706	0.898	0.727	0.960

Source: Authors.

The first-order model showed a good fit to the data (X^2^ = 7,972.988; DF = 335; *p* < 0.001; RMSEA = 0.061; CFI = 0.953; TLI = 0.947) and allowed the direct analysis of the influence of the Physical Environment on the other latent variables. Therefore, it was selected as the most suitable for this analysis.

As shown in Table 6, the Physical Environment (PPE) has a significant and positive influence on all latent variables. Standardized estimates range from 0.502 (95% *CI*: 0.479–0.525, *p* < 0.001) for Personal Wellbeing (PW) to 0.699 (95% *CI*: 0.682–0.716, *p* < 0.001) for Teacher-Student Relationship (TSR).

**Table 6:** Influence of the physical environment on latent variables.

Dependent	Predictor	γ	Lower	Upper	*p*-value
TSR	PPE	0.628	0.606	0.650	<0.001
SSR	PPE	0.699	0.682	0.716	<0.001
LO	PPE	0.621	0.600	0.643	<0.001
COP	PPE	0.588	0.566	0.610	<0.001
PW	PPE	0.502	0.479	0.525	<0.001

Source: Authors.

Physical Environment (PPE) has a significant and positive influence on all latent variables assessed in the study. The standardized estimates range from 0.502 for Personal Wellbeing (PW) to 0.699 for Teacher-Student Relationship (TSR), both of which are statistically significant with a *p*-value less than 0.001. This suggests that as the quality or perception of the Physical Environment improves, there is a corresponding increase in Personal Wellbeing and Teacher-Student Relationship. Specifically, the higher standardized estimate for TSR indicates a stronger relationship between the Physical Environment and the Teacher-Student Relationship compared to Personal Wellbeing.

The confidence intervals (95% *CI*: 0.479–0.525 for PM and 0.682–0.716 for TSR) further reinforce the reliability of these estimates, indicating that we can be confident that the true effect lies within these ranges. In practical terms, these findings imply that enhancing the Physical Environment could lead to improved outcomes in both personal wellbeing and educational settings, particularly in terms of the dynamics between teachers and students. This highlights the importance of considering environmental factors in strategies aimed at improving educational and personal outcomes.

## Discussion and conclusion

The present study aimed to investigate how Chilean teachers’ perceptions of the physical conditions of their classrooms influence their assessments of classroom climate. This exploration is crucial, as the physical environment can significantly impact teaching effectiveness and student engagement (Barrett et al., 2013; Barrett et al., 2015). To achieve this objective, the researchers proposed various analytical models to rigorously test their hypothesis regarding the relationship between the physical environment teachers’ perceptions of classroom climate, and their personal wellbeing. While the second-order model offers a potential solution to address issues of discriminant validity, its inherent analytical complexity and the challenges associated with obtaining stable results suggest that it may not be the most suitable option for Structural Equation Modeling (SEM) analysis in this context. The second-order model often requires intricate data handling and can complicate the interpretation of results, which may detract from the clarity needed for effective communication of findings (Kline, 2023).

In contrast, the first-order model demonstrated an excellent fit and provided easily interpretable results. This model facilitated a direct analysis of the influence of the Physical Environment on other latent variables, such as classroom climate and teacher perceptions. By prioritizing parsimony and clarity, the first-order model aligns well with the study’s objectives, allowing for a straightforward understanding of how physical conditions impact teachers’ assessments. The choice of the first-order model underscores the importance of clarity and simplicity in research design, particularly when exploring complex relationships in educational settings. This approach not only enhances the interpretability of the results but also ensures that the findings can be effectively communicated to stakeholders, such as educators and policymakers, who may benefit from understanding the implications of classroom physical conditions on educational outcomes (Higgins et al., 2012).

Although the second-order model showed similar fit indices, we opted for the first-order specification due to its parsimony and interpretability, which are critical in applied educational research (Lévy and Varela, 2006; Varela et al., 2006; Brown, 2014). In addition, as noted by prior methodological work (Varela et al., 2006; Gould, 2015; Cavicchia and Vichi, 2022), higher-order models can increase estimation complexity and reduce the clarity of substantive interpretations. In our context, adopting a second-order model would also obscure the direct influence of the physical environment (PPE) on each latent factor. While a higher-order specification could simplify the structure by linking PPE to a global construct, it would do so at the expense of evaluating specific influences on classroom climate, wellbeing, and relational variables, which were central to the objectives of this study.

Overall, the study highlights the critical role that the physical environment plays in shaping classroom dynamics and suggests that improving these conditions could lead to more favorable assessments of classroom climate by teachers. This insight emphasizes the need for educational institutions to consider the physical aspects of learning environments as a vital component in fostering positive educational experiences.

The results reveal that physical classroom conditions have significant implications for emotional regulation of both students and teachers. This is in line with previous studies that highlight the interaction between school climate, socio-emotional competencies, and emotional regulation dynamics (Eriksen and Bru, 2023; Ma et al., 2023). In this context, the classroom climate acts as a key mediator that connects the characteristics of the physical space with the emotional and academic outcomes of students.

A crucial aspect of this interaction is how a positive classroom climate can influence students’ emotional regulation by providing a safe and structured environment where self-regulation and emotional expression are actively encouraged. According to Jiménez et al. (2021), elements of emotional support in the classroom, such as support for teachers and collaborative relationships, are directly related to the reduction of antisocial behaviors and the strengthening of socio-emotional skills. In classrooms with poor physical conditions, these supportive dynamics can be compromised, creating a less favorable environment for emotional learning. We note that any references to the physical environment’s potential to promote diversity or to reduce antisocial behaviors are interpretative extrapolations grounded in prior literature, not direct findings of this study.

The study also highlights the relationship between teachers’ wellbeing and their perception of the classroom climate. The results show that aspects such as space, acoustics, cleanliness, and overall comfort in the classroom significantly impact teachers’ motivation and job satisfaction (coefficient of 0.502 for personal wellbeing). This finding aligns with research by Havik and Westergard (2020), which states that teachers’ emotional management directly influences the emotional tone of the classroom and, consequently, students’ experiences. Emotionally balanced and motivated teachers are better equipped to build positive relationships with their students and implement effective strategies to foster emotional regulation.

In addition, the school climate not only affects emotional regulation at the individual level, but also has a collective impact, promoting positive attitudes toward diversity and reducing interpersonal conflicts (Miklikowska et al., 2021). In this sense, an adequate physical environment can facilitate the creation of a space where students feel valued, supported and emotionally safe, contributing to the development of emotional and social competencies. In the Chilean context, where inequalities in infrastructure are evident between public and private schools, these dynamics are especially relevant. Lack of resources and adequate physical conditions in public schools can limit teachers’ ability to establish positive and emotionally enriching classroom climates, perpetuating educational inequalities. Therefore, it is essential to address these gaps from a public policy perspective, promoting equity in school infrastructure and encouraging initiatives that integrate social–emotional education as part of the integral development of students.

From a practical perspective, the findings of this study suggest several lines of action. First, improving school infrastructure should be considered a priority in education policies, especially in highly vulnerable contexts. This includes ensuring basic standards of space, lighting, and temperature that allow both teachers and students to perform in optimal conditions. Second, teacher training programs must incorporate strategies to manage the climate in the classroom and promote emotional regulation, providing teachers with tools to manage complex emotional dynamics in the classroom.

In addition, it is crucial to integrate social–emotional learning approaches into the school curriculum. According to Wang et al. (2020), a positive classroom climate, combined with social–emotional learning strategies, can improve both students’ emotional wellbeing and academic performance. Therefore, classroom design should consider not only physical needs, but also how the environment can facilitate positive interactions and promote emotional development.

We can confirm the importance of physical classroom conditions in shaping teachers’ perceptions of classroom climate and the emotional wellbeing of both teachers and students. These conditions not only affect academic performance, but also have a significant impact on emotional regulation and relational dynamics within the classroom. Improving school infrastructure is not just a matter of material resources, but an investment in the socio-emotional wellbeing of educational actors, which is essential to build inclusive, equitable, and effective learning environments.

A limitation of this study is that the data correspond to the 2018 application of the program. Although it represents a large and nationally representative sample, the age of the dataset may affect the immediate applicability of the findings to current school contexts. It was not possible to include more recent datasets because JUNAEB has not yet anonymized them, which prevents their use in external research.

It should also be noted that, as a cross-sectional and non-experimental study, it is not possible to establish causal relationships between the analyzed variables. In addition, the design may be subject to the influence of confounding variables and potential self-selection bias, which should be considered when interpreting the scope and generalizability of the findings.

Another limitation is that, as a secondary analysis, the researchers had no control over the original instrument design or the sampling process. Moreover, the absence of metadata regarding the sampling methodology makes it difficult to fully assess the representativeness of the data.

It is also important to acknowledge that the original instruments lacked documented psychometric properties, such as prior evidence of reliability or CFA, and presented some inconsistencies in item count. Although these issues were mitigated by the CFA and validity testing conducted in this study, they still represent a limitation when interpreting the results.

It should be noted that handling missing data is a methodological challenge in itself. A central question is how to define what constitutes a “valid participant”: should this be limited to those who respond to all items, or is it acceptable to establish a threshold of answered items that allows for imputation of the remainder? In this study, a 20% missing data threshold was adopted as the exclusion criterion, a decision grounded in methodological references but also in the need to balance rigor with the preservation of the sample. We acknowledge that such decisions involve assumptions that must be considered when interpreting the results and that invite reflection on standard practices in research relying on self-reported data.

Another limitation concerns the reliance on self-reported questionnaires, which may introduce response biases grouped under what the literature terms common method variance (Podsakoff et al., 2003). Although the use of CFA and SEM analyses helped to mitigate these risks through tests of convergent and discriminant validity, it is not possible to rule them out entirely. Future studies could strengthen this aspect by combining self-report data with external observations or complementary indicators, as well as by applying specific statistical techniques to address this type of bias (Yang et al., 2017).

In terms of future projections, it would be valuable to explore how the physical conditions of the classroom interact with other contextual variables, such as the socioeconomic level of school communities or the pedagogical strategies employed. In addition, other research could focus on evaluating the impact of specific interventions, such as infrastructure improvements or the implementation of social–emotional learning programs, on the development of positive school climates. These initiatives would not only help close equity gaps, but also strengthen the education system as a whole, promoting the wellbeing and success of both students and teachers.

Although the findings are strongly tied to the Chilean context, they are consistent with international evidence highlighting the relevance of the physical environment for school climate and educational outcomes (Barrett et al., 2018; Brink et al., 2021). This suggests that the results may offer useful insights for other education systems facing comparable inequalities in infrastructure and resources, while recognizing the particularities of each setting.

## Data Availability

The dataset analyzed in this study was provided by the Junta Nacional de Auxilio Escolar y Becas (JUNAEB), Chile, through Resolución Exenta Nº DN-02620/2024. The data were delivered fully anonymized. Researchers may request access directly from JUNAEB, in accordance with the Chilean Transparency Law (Law No. 20,285). In addition, a derived dataset resulting from the imputation of missing data, corresponding to the analyses conducted in this study, is openly available at https://doi.org/10.7910/DVN/ZWR1R2.
